# Mitochondrial DNA analysis of critically endangered Chinese Pangolins (*Manis pentadactyla*) from Nepal

**DOI:** 10.1080/23802359.2020.1811174

**Published:** 2020-08-31

**Authors:** Sandeep Shrestha, Ashish Bashyal, Ashna Dhakal, Thomas J. McGreevy, Bill Buffum, Jyoti Joshi, Hemanta Kumari Chaudhary, Sanjay Nath Khanal

**Affiliations:** a Department of Environmental Science and Engineering, Kathmandu University, Kavrepalanchok, Nepal; b Biodiversity Conservancy Nepal, Butwal, Nepal; c Department of Biotechnology, Kathmandu University, Kavrepalanchok, Nepal; d Department of Natural Resources Science, University of Rhode Island, Kingston, RI, USA; e Center for Molecular Dynamics, Kathmandu, Nepal

**Keywords:** COI, DNA barcode, illegal trade, Pangolin

## Abstract

Chinese Pangolins (*Manis pentadactyla*) are Critically Endangered and one of the most illegally traded mammals globally. We generated first COI sequences from five individuals of this species from Nepal. BLASTn search of our 600 bp sequences at GenBank showed pair-wise identity between 99.17% and 100% to *M. pentadactyla*. There were three haplotypes and a total of five variable sites among five *M. pentadactyla* sequences. Neighbor-joining tree revealed that all *M*. *pentadactyla* from Nepal clustered into same group further splitting into two sub-groups albeit with low bootstrap value, suggesting potential multiple geographic origins. The K2P distance was 0.3% within group and 0.7% between four sequences from Bhaktapur and Kavrepalanchok districts (Mape2, Mape3, Mape5 and Mape6) and museum sample (Mape10). This study has generated reference samples for *M. pentadactyla* from Nepal and will be helpful in understanding dynamics of illegal trade of this species and in successful identification of *M. pentadactyla* from Nepal even in the absence of intact specimens.

## Introduction

Chinese Pangolins (*Manis pentadactyla*) are one of the most globally threatened mammals, primarily due to overexploitation by the illegal international wildlife trade and local consumption (Challender et al. 2019). Chinese Pangolins are ‘Critically Endangered’ and listed on Appendix I of CITES (Convention on International Trade in Endangered Species of Wild Flora and Fauna) (CITES, 2017; Challender et al. 2019). They are widely distributed in Asia from China in the east to Nepal in the west. They occur in 27 districts in Nepal across the eastern, central and western regions of the country, within and outside protected areas and are confined to an altitude between 1500 − 1844 meters (m) above sea level (DNPWC, 2019; Suwal et al. 2020; Wu et al. 2020). The illegal trade in Chinese Pangolins has been fueled by demand for its meat for consumption as a delicacy and for its scales and other body parts for use in traditional medicine (Challender et al. 2019). Between 2000 and 2013, approximately 50,000 Chinese Pangolins were involved in international trafficking (Challender et al. 2015). In the eastern part of Nepal, Chinese Pangolins scales are traded by local hunters at the rate of $7–12.5 per kg and the price rises as scales reach international markets (Katuwal et al. 2015).

The main challenge, after the confiscation of pangolin scales and body parts, is to confirm the species identity of the scales (Zhang et al. 2015; Luczon et al. 2016). For a wide ranging and highly trafficked species such as the Chinese Pangolin, having reference mitochondrial DNA cytochrome c oxidase subunit 1 (COI) sequences from different geographic regions throughout its distribution would be helpful not only to identify the species of confiscated samples, but also to delineate the geographic origin of the samples. There have been few molecular genetic studies conducted on DNA derived from Chinese Pangolin scales (Hsieh et al. 2011; Zhang et al. 2015; Luczon et al. 2016) and no studies to date have been carried out in Nepal. To address the critical need for additional COI sequences for Chinese Pangolins from different parts of their geographic range, we collected Chinese Pangolin scales found during our field surveys in different community forests of Nepal. We generated the first COI sequences for Chinese Pangolin samples from known geographic locations in Nepal.

## Materials and methods

### Sample collection

**Figure 1.**
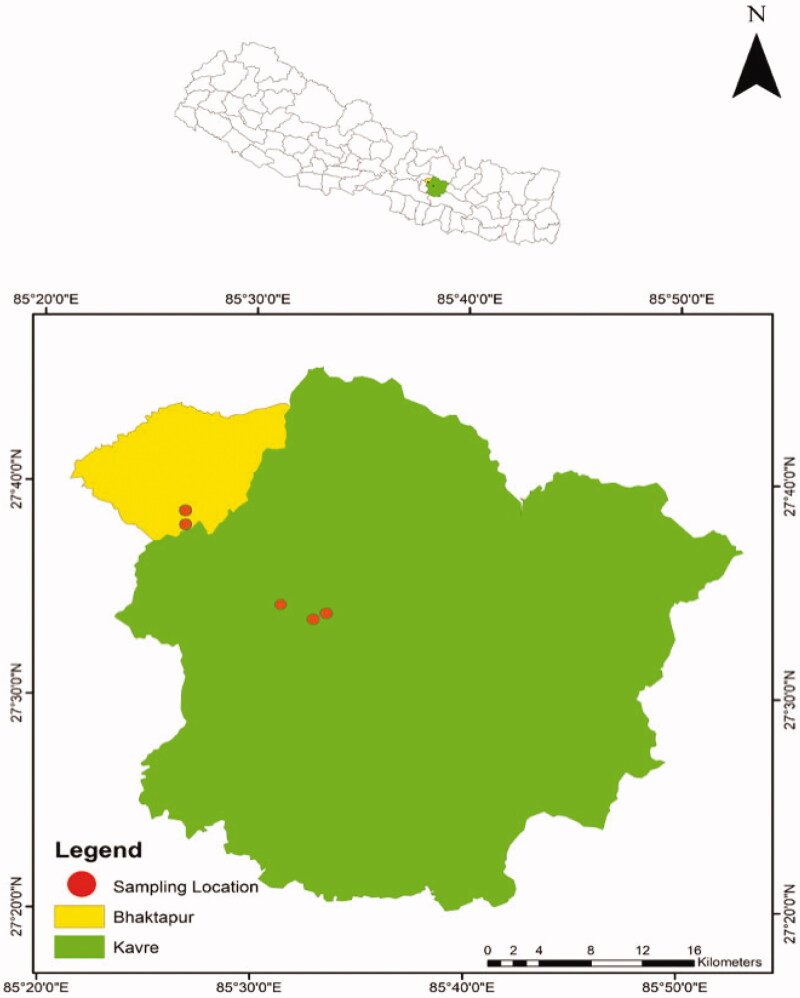
Map of Nepal (inset) indicating tentative locations of sampled community forests in Bhaktapur and Kavrepalanchok districts. Names and exact locations of community forests are not disclosed for safety reasons.

During our ongoing ecological study on Chinse Pangolins in mid-hill regions of Nepal between December 2017 and April 2019, we collected nine pangolin scales from various community forests in Bhaktapur and Kavrepalanchok Districts located in Province 3 of Nepal (Figure 1). We found all these scales in isolation and not from the animal/carcass itself. Names and locations of community forests are not disclosed for safety reasons. We also obtained one sample (tissue) stored in formalin from the Natural History Museum of Nepal; the date of collection and geographic origin of this sample was not available. We cleaned the scale samples with distilled water and stored them at 4 °C in new Ziploc bags until they were processed for genetic analyses.

### DNA extraction, PCR amplification and sequencing

Genetic laboratory work and gene sequencing was performed by the Center for Molecular Dynamics Nepal (CMDN). Scales were crushed into powder using a sterile mortar and pestle and total genomic DNA was extracted using Gene All Tissue mini kit (Gene All ExgeneTM Tissue SV kit) following manufacturer’s instruction. A partial fragment of the COI was amplified using primer set LCO1490 (5′-GGTCAACAAATCATAAAGATATTGG-3′) and HCO2198 (5′-TAAACTTCAGGGTGACCAAAAAATCA-3′) (Folmer et al. 1994). Polymerase chain reactions (PCR) were performed in a 25 µl volume, which contained 12.5 µl multiplex master mix (Qiagen, Germany), 5 µl Q solution (Qiagen, Germany), 4.5 µl of RNAse free water (Qiagen, Germany), 1 µl (10 mM) of each forward and reverse primer and 1 µl of undiluted DNA template. Thermocycling conditions followed Hebert et al. (2003) and consisted of one cycle of 1 min at 94 °C; five cycles of 1 min at 94 °C, 1.5 min at 45 °C and 1.5 min at 72 °C; 35 cycles of 1 min at 94 °C, 1.5 min at 50 °C and 1 min at 72 °C; and a final cycle of 5 min at 72 °C. Samples were PCR amplified using a PTC-225 Peltier Thermal Cycler (MJ Research Inc.) and products were visualized by electrophoresing the amplicons through a 2% agarose gel using a Gel-Doc (Major Scientific TM) system.

PCR products were cleaned using enzymatic clean up (ExoSAP-IT). Sequencing reactions were performed using an ABI PRISM^®^ BigDyeTM Terminator V 4.1 kit with AmpliTaq^®^ DNA polymerase (FS enzyme) (Applied Biosystems), following the protocols supplied by the manufacturer. Fluorescent-labeled fragments were purified from the unincorporated terminators using the BigDye^®^ XTerminator™ purification protocol. The samples were re-suspended in distilled water and subjected to electrophoresis in an ABI 3730xl sequencer (Applied Biosystems). Sequencing of the PCR products were performed in both the forward and reverse directions.

### Phylogenetic analysis

**Figure 2.**
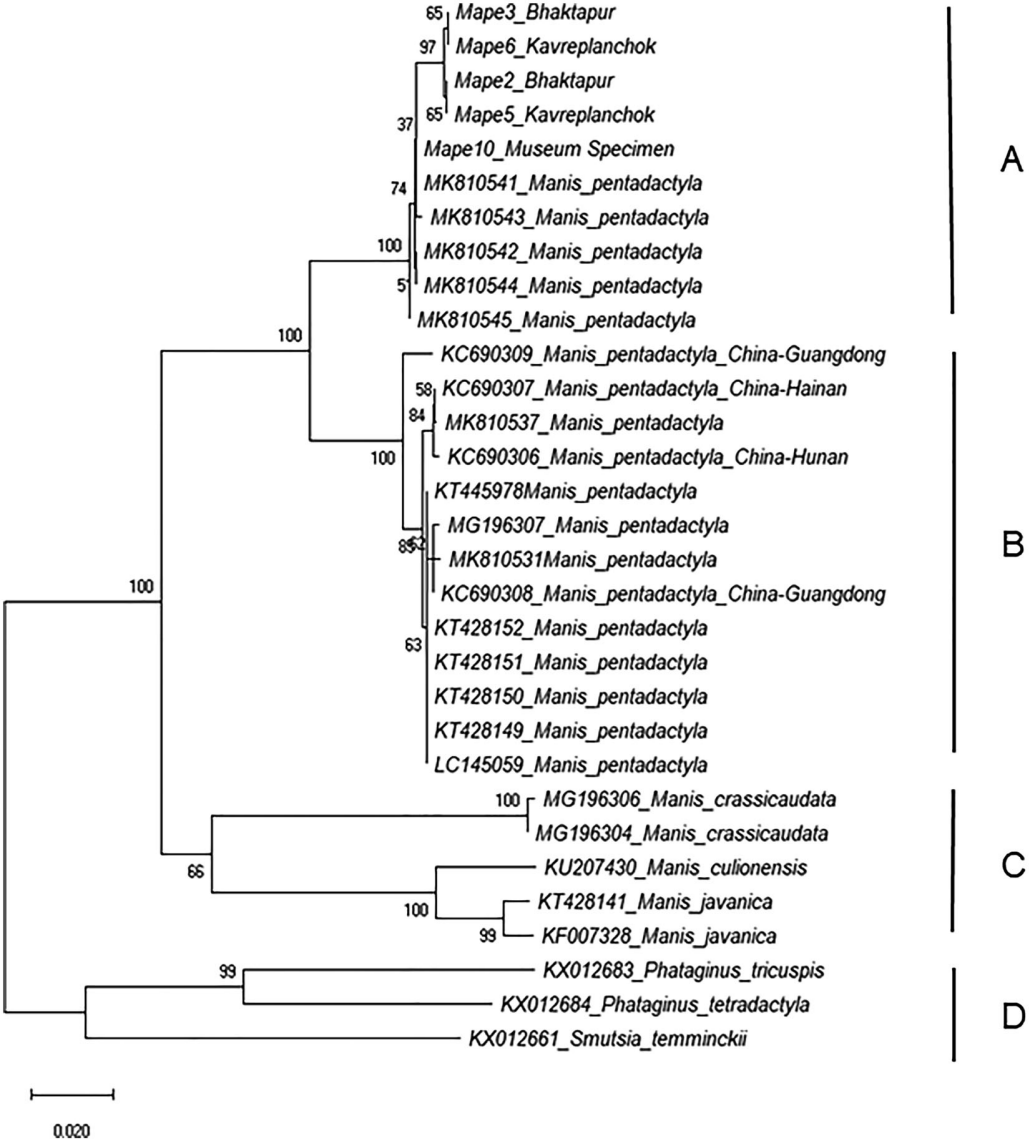
Unrooted mitochondrial DNA subunit c cytochrome oxidase subunit 1 neighbor-joining tree based on a Kimura-2-Parameter (K2P) model of evolutionary change. The tree shows the relationships of *Manis pentadactyla* samples from Nepal with *M. pentadactyla* samples from China and unknown origins as well as with other Asian and African pangolin species downloaded from GenBank (taxa with accession numbers). The NJ tree has four major groups – A = *M. pentadactyla* group from Nepal and unknown origins, B = *M. pentadactyla* group from China and unknown origins, C = Asian pangolin group, and D = African pangolin group. Values at node represent bootstrap values from 500 replicates. Scale bar represents two nucleotide substitutions for every 100 nucleotides. Geographic location of origin of samples when available are mentioned following species name for sequences acquired from the GenBank.

We aligned forward and reverse sequences in Geneious 9.1.8 and trimmed to uniform length of 600 nucleotides. We accessed GenBank to acquire 18 *M. pentadactyla* sequences to have as extensive representation of the species as possible. We acquired two sequences each for *M. javanica* and *M. crassicaudata* and one sequence each for *M. culionesis*, *Phataginus tricuspis*, *P. tetradactyla* and *Smutsia temminckii*. We have provided accession number for all 26 sequences acquired from GenBank in Figure 2. We aligned all 31 sequences using the Clustal W algorithm in MEGA X (Kumar et al. 2018). We used a Kimura-2-Parameter (K2P) (Kimura 1980) model of evolutionary change and constructed an unrooted Neighbor-joining tree (Saitou and Nei 1987) in MEGA X (Figure 2).

## Results and discussion

We obtained COI sequences from six samples (two from Bhaktapur District, three from Kavrepalanchok District, and one from the Natural History Museum). We discarded one sequence from Kavrepalanchok District due to it’s low quality. Standard Nucleotide Blast (BLASTn) search at GenBank (http://blast.ncbi.nlm.nih.gov/) employing blastn algorithm for all five sequences showed pair-wise identity between 99.17% and 100% to *M. pentadactyla* (Table 1). There were three haplotypes (Mape 3 & Mape 6; Mape 2 & Mape 5; and Mape 10) among five *M. pentadactyla* sequences. Similarly, there were a total of five variable sites among 600 bp and interestingly four of those were in Mape 10.

**Table 1.** Output from BLASTn showing query cover, pairwise identify, species and accession numbers of COI sequences for top hits against *M.pentadactyla* sequences from this study.

Sequence ID	Accession number for sequence from this study	Query cover (%)	Pairwise identity (%)	Accession number	Species
Mape 2	MT712655	100	99.33	MK810544; MK810541; MK810542	*M. pentadactyla*
Mape 3	MT712656	100	99.17	MK810544; MK810541; MK810542; MK810545	*M. pentadactyla*
Mape 5	MT712657	100	99.33	MK810544; MK810541; MK810542	*M. pentadactyla*
Mape 6	MT712658	100	99.17	MK810544; MK810541; MK810542; MK810545	*M. pentadactyla*
Mape 10	MT712659	100	100	MK810544; MK810541; MK810542	*M. pentadactyla*

Neighbor-joining tree constructed from 31 COI sequences (five from this study and 26 acquired from GenBank), revealed four major groups (A–D; Figure 2). Group A included *M*. *pentadactyla* from Nepal and unknown origins. *Manis pentadactyla* from Nepal split into different sub-groups albeit with low bootstrap value suggesting potential multiple geographic origins. *Manis pentadactyla* from Kavrepalanchok and Bhaktapur districts grouped together in one sub-group whereas the other sub-group included the *M. pentadactyla* sample acquired from the Natural History Museum of Nepal (the geographic origin of this sample is unknown) along with *M. pentadactyla* sequences acquired from GenBank with unknown geographic origins. Three sequences (MK810544; MK810541; MK810542) have 100% pair-wise identity with Mape10 (Table 1); however, due to lack of information on geographic origins of these sequences, no inferences could be made on such placement. Group B included *M. pentadactyla* from China (KC690306, KC690307, KC690308, and KC690309) and unknown origins. Group C formed a distinct group of three Asian species of pangolins (*M. crassicaudata*, *M. javanica* and *M. Culionesis*) and group D formed a distinct group of three African pangolin species (*Phataginus tricuspis*, *P. tetradactyla* and *Smutsia temminckii*). The lowest K2P distance (5.8%), as expected, was between two *M. pentadactyla* groups (A and B) and the highest (23.9%) was between Group C (Asian pangolin species) and Group D (African pangolin species) (Table 2). Genetic distance was only 0.3% within five sequences of *M. pentadactya* from Nepal, 0.1% within four sequences (Mape2, Mape3, Mape5, Mape6) and 0.7% between four sequences from two districts (Mape2, Mape3, Mape5, Mape6) and museum sample (Mape10).

**Table 2.** Pairwise genetic distance between groups (A–D) based on Kimura 2 Parameter model of evolutionary change.

Groups	A	B	C	D
A	0			
B	0.058	0		
C	0.162	0.149	0	
D	0.211	0.232	0.239	0

Efficacy of COI in resolving pangolin phylogeny has already been demonstrated (Zhang et al. 2015; Luczon et al. 2016; Mwale et al. 2017). We generated the first record of COI sequences for *M. pentadactyla* from Nepal and demonstrated its efficacy in delineating *M. pentadactyla* samples to multiple geographic origins. Splitting of *M. pentadactyla* samples from China and Nepal into two distinct groups with high bootstrap value (100%) demonstrates its ability to distinguish among samples from different countries. Furthermore, presence of three haplotypes within five samples in our study and splitting of *M. pentadactyla* from Nepal (Group A) into two sub-groups demonstrates that this gene is potentially able to distinguish samples from different geographic origins within a country. For instance, the *M. pentadactyla* sample from the Natural History Museum of Nepal was in a different sub-group than the four samples from Kavrepalanchok and Bhaktapur district, which are in close proximity to each other (Figure 2). The successful analysis of this museum sample has also opened the possibility of creating a reference data base for *M. pentadactyla* for Nepal using available museum specimen.

Approximately 94% of potential *M. pentadactyla* habitat covering 28,768 km^2^ in the mid-hill region of Nepal occur outside protected areas (Sharma et al. 2020). Poaching is one of the major threats for the species in Nepal and pangolin scales are confiscated in the country regularly (Katuwal et al. 2015). This study has generated reference samples for *M. pentadactyla* from Nepal and will be helpful in understanding dynamics of illegal trade of this species and in successful identification of *M. pentadactyla* from Nepal even in the absence of intact specimens. We recommend building a comprehensive reference database for COI sequences from *M. pentadactyla* samples from wider geographic range including eastern and western limits. Such a database could potentially delineate the geographic origin of samples from Nepal; thus, providing information on locations where poaching is happening, which would greatly help authorities take action to counter poaching.

## Data Availability

We collected samples under Permit no: 53- 2074/2075 issued by the Department of Forests and Soil Conservation, Nepal. Five COI sequences for *M. pentadactyla* from this study are deposited at the GenBank (Accession numbers: MT712655, MT712656, MT712657, MT712658 and MT712659). Genbank: https://www.ncbi.nlm.nih.gov/genbank/ where our data has been stored.
